# Cryo-EM analysis of Pseudomonas phage Pa193 structural components

**DOI:** 10.21203/rs.3.rs-4189479/v1

**Published:** 2024-04-12

**Authors:** Gino Cingolani, Stephano Iglesias, Chun-Feng Hou, Sebastien Lemire, Angela Soriaga, Pierre Kyme

**Affiliations:** University of Alabama at Birmingham; Thomas Jefferson University

**Keywords:** Pbunavirus, Pseudomonas-phages, tail fiber, cryo-EM, bacterial adhesion and genome ejection, cystic fibrosis, phage therapy

## Abstract

The World Health Organization has designated *Pseudomonas aeruginosa* as a critical pathogen for the development of new antimicrobials. Bacterial viruses, or bacteriophages, have been used in various clinical settings, commonly called phage therapy, to address this growing public health crisis. Here, we describe a high-resolution structural atlas of a therapeutic, contractile-tailed *Pseudomonas* phage, Pa193. We used bioinformatics, proteomics, and cryogenic electron microscopy single particle analysis to identify, annotate, and build atomic models for 21 distinct structural polypeptide chains forming the icosahedral capsid, neck, contractile tail, and baseplate. We identified a putative scaffolding protein stabilizing the interior of the capsid 5-fold vertex. We also visualized a large portion of Pa193 ~ 500 Å long tail fibers and resolved the interface between the baseplate and tail fibers. The work presented here provides a framework to support a better understanding of phages as biomedicines for phage therapy and inform engineering opportunities.

## INTRODUCTION

Infections caused by the Gram-negative pathogen *Pseudomonas aeruginosa* are a leading cause of morbidity and mortality worldwide. *P. aeruginosa* is a significant public health concern because many strains have acquired antibiotic-resistance genes, and the bacterium forms biofilms impermeable to many antibiotics and refractory to common antimicrobials^1^. *P. aeruginosa* infections are particularly significant in cystic fibrosis (CF) patients. CF is a multiorgan disease caused by mutations in the Cystic Fibrosis Transmembrane Conductance Regulator (CFTR) gene that encodes a membrane protein channel regulating chloride and bicarbonate ion transport in pulmonary epithelia. The altered function of the CFTR protein causes electrolytic imbalance and dehydration of the airway’s surface, leading to increased mucin concentration and defective mucociliary clearance of microbial pathogens^2,3^. The pathophysiology of CF includes recurrent bacterial infections and persistent inflammation, with the microbial community mainly composed of *Staphylococcus aureus* at a young age and *P. aeruginosa* later in life^4^. Over time, *P. aeruginosa* acquires specific mutations and adaptive responses to antibiotic exposure, eventually leading to the selection and diffusion of multidrug-resistant (MDR) strains^5^. The eradication by antimicrobial treatment is complicated by intrinsic bacterial resistance to numerous antibiotics. Phage therapy, especially against *P. aeruginosa*, has gained attention as a promising therapeutic weapon in the fight against CF-related infections^6,7^.

Pa193 is a *P. aeruginosa* lytic bacteriophage of the *Myoviridae* superfamily, *Pbunavirus* genus, which is characterized by a long contractile tail and a small baseplate, similar to the *Pseudomonas* phage E217^8^ and cyanophage Pam3^9^. Pa193 has a double-stranded DNA (dsDNA) genome of ~ 66.7 kbp, slightly larger than ~ 195 *Pbunaviruses* deposited in the NCBI database, that encodes 96 Open Reading Frames (ORFs). Pa193 is significantly smaller than T4, a classical *Myoviridae* coliphage^10–12^, whose ~ 168.9 kbp genome encodes about 300 gene products, over three times the size and complexity of *Pseudomonas-*phage Pa193. The baseplate is arguably T4’s most complex component, shared by all *Myoviridae*. This multisubunit complex is vital to the phage’s most critical activities: tail assembly, host attachment, host outer and inner membrane (IM) penetration, and contraction-coupled genome ejection. The architecture of the T4 isolated baseplate has been elucidated in great detail, both in the contracted and extended conformation of the phage tail^12–14^. The T4 baseplate is built by 15 different polypeptide chains generating a ~ 6 MDa machine that harbors two sets of six fibers, known as short- and long-tail fibers, responsible for phage attachment to the bacterial cell wall^15,16^. Cryo-electron tomography (cryo-ET) studies of T4 infecting cells^17^ revealed that T4 long-tail fibers fold back against the virion before infection and do not interact directly with the host surface. The short tail fibers that protrude from the baseplate bottom are instead responsible for host outer membrane attachment, which triggers contraction. Remarkably simpler than T4 is the baseplate from the *Pseudomonas* phage E217 (~ 1.4 MDa in mass)^8^. E217 exerts the same essential function of host attachment and signal transduction as T4, which initiates tail contraction, membrane penetration, and sheath-contraction-coupled genome ejection. E217 baseplate closely resembles the R-type pyocin baseplate^18–20^, which comprises six highly flexible fibers connected laterally to the baseplate that, upon receptor-binding, initiate a cascade of events that lead to sheath contraction^21^. Notably, the E217 baseplate is also different than Twort-like *Myoviridae* phages like phi812^22^ and SaGU1^23^, whose baseplate proteins are organized into two layers that separate after tail contraction.

In this study, we describe the atomic structure of the *Pseudomonas* phage Pa193, which we solved using the power of cryo-EM Single Particle Analysis (SPA) and localized reconstruction, together with proteomics and bioinformatics.

## RESULTS

### Atomic structure of Pseudomonas phage Pa193

**Figure 1:**
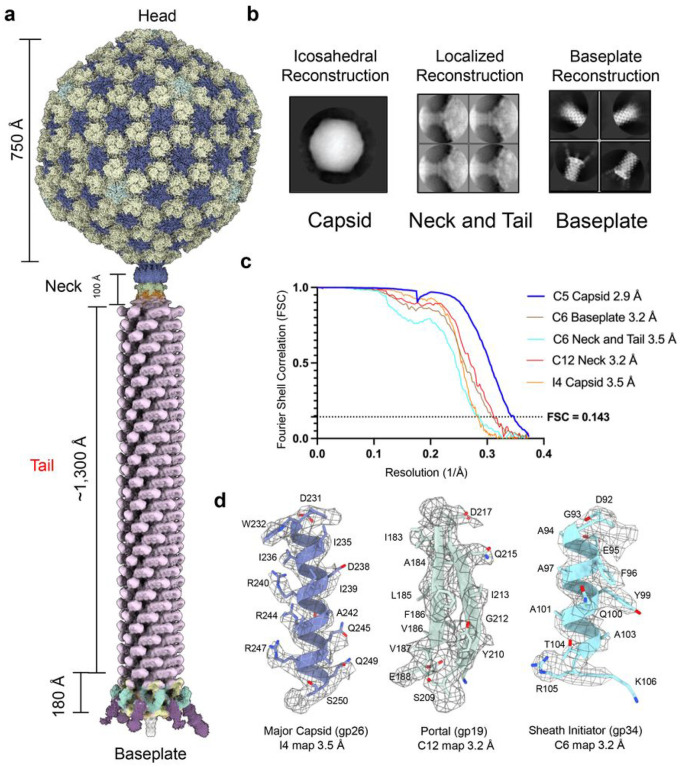
Cryo-EM reconstruction of *Pseudomonas* phage Pa193. (**a**) Composite map of the Pa193 virion with an extended tail. (**b**) 2D class averages of each region of Pa193 and the corresponding SPA method used: (left) the capsid solved using icosahedral (I4 symmetry) reconstruction; (center) the neck and tail determined using localized reconstruction; (right) the baseplate determined picking the tail tip distal from the capsid with C6 symmetry imposed. (**c**) Fourier Shell Correlation (FSC) curves for each reconstruction used in this paper. (**d**) Representative densities corresponding to each cryo-EM SPA technique above. (Left) Major capsid protein residues 231–250 (purple) fit in the I4 icosahedral reconstruction; (Middle) Portal protein residues 183–188; 209–217 (green) fit into the C12 localized reconstruction; (Right) Sheath initiator protein residues 92–106 (light blue) fit in the C6 baseplate reconstruction.

**Figure 2:**
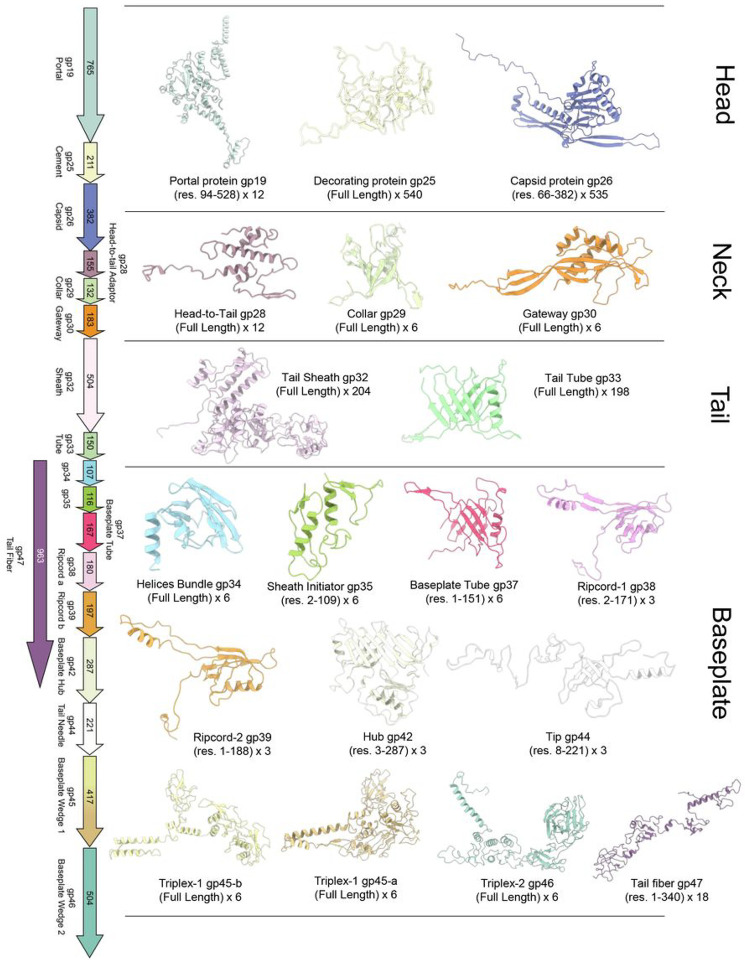
Structural atlas of *Pseudomonas* phage Pa193. Ribbon diagrams of 19 of the 21 gene products identified and built *de novoin* this work: three in the capsid, four in the neck, three in the tail, and eleven in the baseplate. 19 ORFs were annotated and built as full-length and are shown in Figure 2. A few residues were modeled for the putative scaffolding, gp24 (not shown in Fig. 2; see Fig. 4 and tape measure protein, gp41 (not shown in Fig. 2; see Fig. 9

We used cryo-EM SPA to reconstruct *Pseudomonas* phage Pa193 in the extended tail state (Table 1, Fig. 1a). We reconstructed the entire bacteriophage using focused maps of each region of the phage: head, neck/tail, and baseplate (Fig. 1a, b). First, we generated a 3.5 Å reconstruction of the capsid while imposing icosahedral symmetry (I4) (Fig. 1c). Then, we used localized reconstruction to generate a C5 2.9 Å reconstruction of the capsid five-fold vertex (Fig. 1c, **Supplementary Fig. S1**), which allowed us to build the major capsid and decorating proteins. The neck, composed of collar, gateway, tube, and sheath proteins, was similarly reconstructed using localized reconstruction by selecting a class of particles with tails at one five-fold vertex and imposing C6 symmetry, which yielded a 3.5 Å map (Fig. 1 c, **Supplementary Fig. S1**). We then built the dodecameric, portal, and head-to-tail proteins by imposing C12 symmetry on the same particles, yielding a 3.2 Å map. Finally, we reconstructed the baseplate by manually picking 40,335 baseplate particles and applying (C6) symmetry to obtain a 3.2 Å reconstruction (Fig. 1c, **Supplementary Fig. S2**). In all cases, the reconstructions had excellent side-chain density (Fig. 1d), which allowed us to annotate, *de novo* build, and refine 19 full-length phage Pa193 ORFs (Fig. 2) and two partial ORFs real-space refined to a map-to-model correlation coefficient (CC) greater than 0.8 (Table 2). Overall, Pa193’s structural atlas presented in this paper (Fig. 2) comprises three capsid proteins (gp19, gp25, and gp26), three neck proteins (gp28, gp29, and gp30), two tail proteins (gp32 and gp33), and 11 baseplate proteins (gp34, gp35, gp37, gp38, gp39, gp42, gp41, gp44, gp45, gp46 and gp47), highlighting the structural complexity of this *Myoviridae*.

### Architecture of Pseudomonas phage Pa193 T = 9 capsid

**Figure 3:**
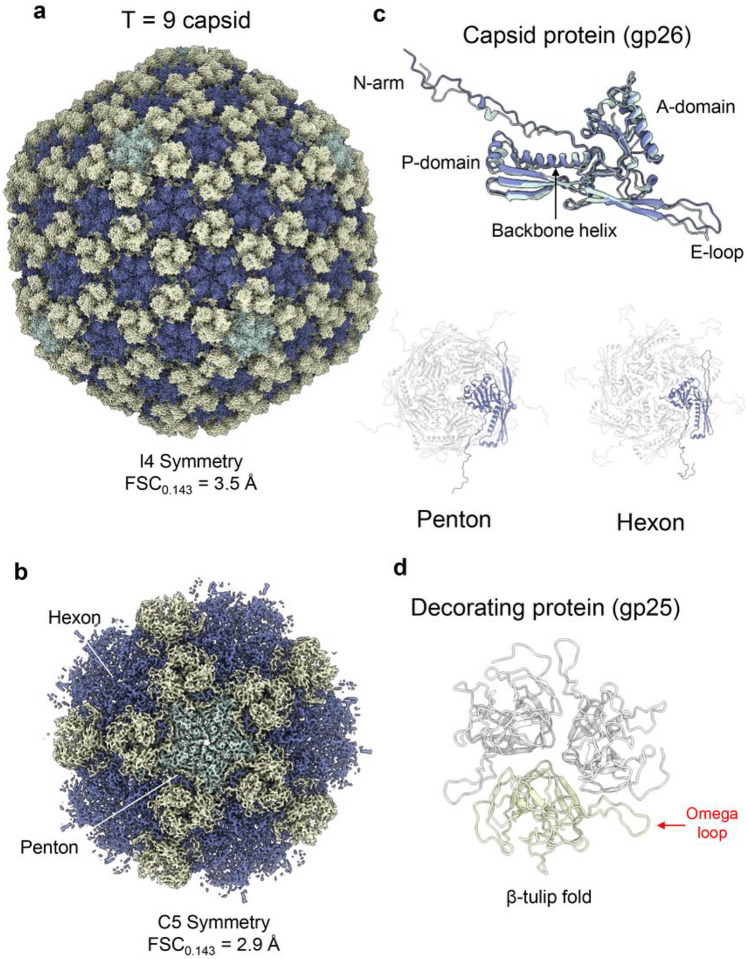
Identification and annotation of Pa193 phage capsid and decorating proteins. (**a**) Cryo-EM reconstruction of Pa193 capsid measured at 3.5 Å resolution and displayed at 3.5 σ. Major capsid protein assembles as pentons are colored (green) or hexons (blue), and decorating protein forms a trimer at every three-fold vertex (yellow). (**b**) Cryo-EM localized reconstruction of Pa193 capsid five-fold vertex at 2.9 Å resolution. The map is displayed at 4.5 σ and has the same protein color scheme as in panel (**a**). (**c**) *(Top)* Overlay of the Pa193 capsid protein gp26 conformations found at the penton and hexon (RMSD 1.08 Å). (*Bottom*) Ribbon diagrams of the capsid protein assembled in a penton *(left)* or as part of a hexon *(right)*. (**d**) Ribbon diagram of the decorating protein gp25 (residues 1–211) assembled as a trimer.

An I4 icosahedral reconstruction of the Pa193 capsid at 3.5 Å resolution (Fig. 3a) and a 2.9 Å focused reconstruction of a capsid five-fold vertex (Fig. 3b) revealed two components of Pa193 head: the major capsid protein gp26 (residues 66–382) (Fig. 3c) and the decorating protein gp25 (residues 1–211) (Fig. 3d). Pa193 head is about 750 Å wide, 25 MDa, built by 535 copies of the 41.6 kDa gp26 that forms a lattice with a triangulation number T = 9. Gp26 (Fig. 3c) adopts a canonical HK97 fold that comprises an E-loop (residues 126–140), Proximal domain (P-domain; residues 159–176; 352–357), N-arm (residues 66–96), backbone helix (residues 175–197), and Axial domain (A- domain; residues 216–320, 372–382)^24^. Pa193 capsid protein conforms to the *quasi*-equivalence theory of viral capsids^25^, with the same protein in two quasi-equivalent conformations assembled into hexons or pentons (Fig. 3c) of different diameters (hexon = 190 Å vs. penton = 157 Å) and quaternary structure, where pentons appear raised or convex relative to the plane of the capsid (Fig. 3c).

The second protein identified in Pa193 capsid reconstruction, gp25, is a trimer similar to the E217 decoration protein gp26 (Fig. 3d). One hundred eighty copies of Pa193 gp25 (M.W. 21.6 kDa) assembled as sixty trimers bind the capsid exterior at each three-fold vertex (Fig. 3a). Each trimer contacts three neighboring decorating proteins and six capsid protein subunits from three adjacent capsomers. This pattern generates a second capsid layer decorating the exterior surface that buries a surface area of 8,164 Å^2^. Gp25 trimers at neighboring three-fold/quasi-three-fold axes generate a cage surrounding the capsid protein shell (Fig. 3a). The extensive pattern of van der Waals contacts between gp26 protomers N-terminal arms and omega loops (Fig. 3d) suggests that the interconnected architecture of gp25 trimers stabilizes the capsid. Thus, the decoration protein gp25 likely functions like a cementing protein, structurally similar to lambda gpD^26^.

### A helical protein binds the capsid interior at five-fold vertices

**Figure 4:**
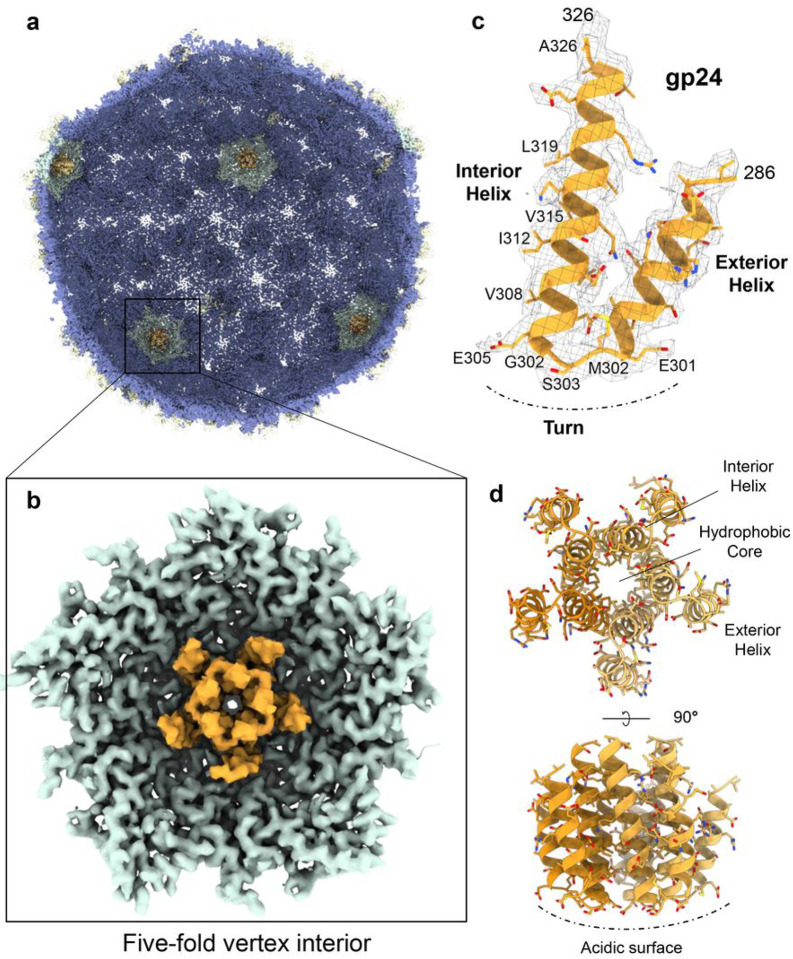
Identification of Pa193 putative scaffolding protein gp24. (**a**) Cross-section view of Pa193 capsid map (I4 symmetry). (**b**) magnified view of a five-fold vertex visualized from the capsid interior, highlighting the helix-turn-helix density visible at the five-fold vertex (colored orange). (**c**) Magnified view of the putative scaffolding protein density calculated at 3.5 Å resolution and displayed at 1.5 σ, overlaid to the Model Angelo model. (**d**) Cartoon representation of the gp24 pentamer.

Attentive analysis of the I4 and C5 maps revealed a unique density in the capsid interior at each of the five-fold vertexes (Fig. 4a). To identify the ORF encoding this factor, we first built a poly-alanine model into the density that consists of a pentameric assembly of a helix-turn-helix motif (Fig. 4b). Each protomer comprises a long Interior Helix involved in oligomerization and an outward facing Exterior Helix (Fig. 4b, c). We hypothesized this helical assembly to be a remnant of a putative scaffolding protein, possibly cleaved after assembly. Consistent with this hypothesis, the helix-turn-helix motif has been observed in the scaffolding protein of other bacteriophages like P22 (PDB: 2GP8^27^) and φ29 (PDB: 1NOH; 1NO4;^28^). Next, we searched the Pa193 genome to identify the ORF encoding a scaffolding protein. There are five unaccounted ORFs in the proximity of Pa193 capsid, neck, and tail proteins, namely gp20, gp21, gp24, gp27, and gp31, whose gene products were identified by liquid chromatography-mass spectrometry (LC-MS) in the mature Pa193 virion (**Supplementary Table S1**). We used Model Angelo^29^ to build an *ab initio* model that included side chains, resulting in five identical models making up a pentameric bundle. Sequence alignments of the Model Angelo predicted sequences against the candidate ORFs yielded a consistent match to gp24 residues 286–326. ORF24, located in the proximity of the gene encoding the decorating protein gp25 and capsid protein gp26 (Fig. 2) and possibly expressed at the same time, encodes a gene product, gp24, that may function as a scaffolding protein for capsid assembly. Val308, Ile312, Val315, Leu319, Leu322, and Ala326 in the interior helix (Fig. 4c) stabilize the gp24 pentamer (Fig. 4d) that inserts at the five-fold vertex like a dowel pin (Fig. 4b).

Next, we focused on the binding interface between the Pa193 capsid proteins and the gp24 putative scaffolding fragment. The gp24 helix-turn-helix fragment identified in our reconstruction contains a negatively charged turn (residues 301–305, sequence EMSGE) (Fig. 3c); five turns form an acidic surface in the pentamer facing the five-fold vertex in the capsid (Fig. 4d). An electrostatic surface potential map of the capsid protein interior (**Supplementary Fig. S3a**) reveals that capsid protein residues Arg295 and Lys293 make two salt bridges with gp24-Glu301 and gp24-Glu310, respectively (Fig. 4c, d **and Supplementary Fig. S3b, c**). In addition, the capsid protein gp26 makes four additional hydrogen bonds with mainchain and side chains atoms of gp24, including gp26-Pro292:gp24-Ser303, gp26-Tyr291:gp24-Ser303, and gp26-Glu301:gp24-Ile296 (**Supplementary Fig. S3b, c**). The binding interface between pentameric gp24 and the capsid interior five-fold vertex is 2,974.6 Å^2^, comprising 20 salt bridges and 30 hydrogen bonds. Thus, we hypothesize that gp24 is a putative scaffolding protein that persists in the mature virion by directly stabilizing the capsid five-fold vertices.

### The neck of Pseudomonas phage Pa193

**Figure 5:**
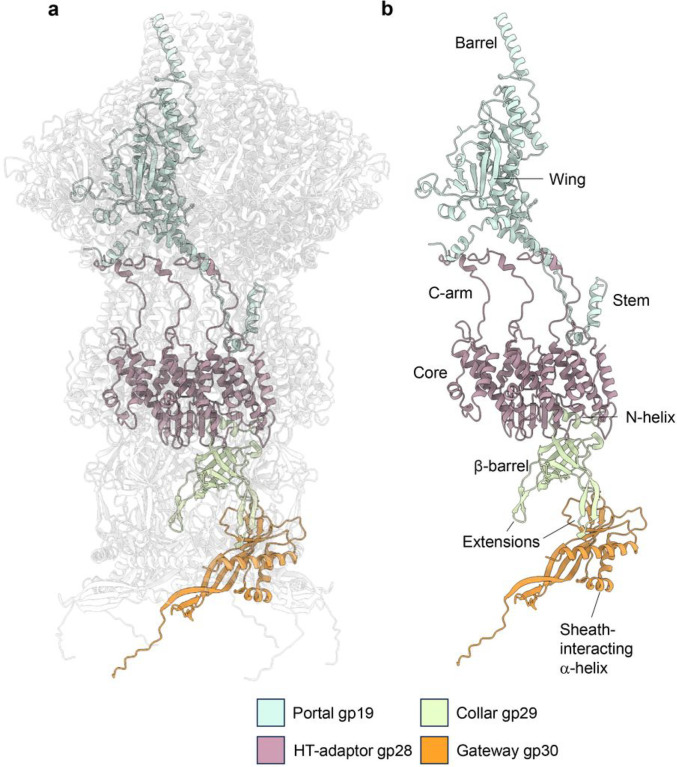
Structure of Pa193 neck assembly. (**a**) Overview of the Pa193 neck complex. Portal gp19 (green) binds to the HT-adaptor gp28 (purple), which in turn binds to the collar gp29 (light green) and gateway gp30 (orange). (**b**) Ribbon diagram of the isolated neck protomer: gp19 (residues 94–528), gp28 (residues 1–155), gp29 (residues 1–132), and gp30 (residues 1–183).

We also generated two high-resolution symmetric reconstructions, a C12 map and a C6 map of phage Pa193 neck proteins. The C12 map was used to identify and build *de novo* models of the portal gp19 and head-to-tail gp28 complexes (Fig. 5a). The neck is the attachment point for the ~ 1,300 Å long tail and 180 Å wide baseplate. Pa193 portal and head-to-tail proteins assemble as a dodecameric complex and are positioned at one of the 12 five-fold vertices of the capsid. During phage assembly, this complex is a docking site for genome packaging proteins called terminases, which package a dsDNA genome into the capsid^30^. The portal also contacts major capsid proteins with a 12:10, symmetry-mismatched binding interface^31^. Pa193 portal structure was built in a 3.2 Å map (Fig. 1c) and lacks density for the first 93 amino acids. These residues were not identified by LC-MS and may be cleaved during maturation or are invisible in the reconstruction due to the symmetry mismatch between portal and capsid proteins^32^. Pa193 portal is most similar to E217 portal protein gp19 (RMSD 1.38 Å) and presents a classical portal protein fold including a barrel, wing, and stem domains (Fig. 5b)^31^. The C-terminal barrel has about 30 residues in our reconstruction, and intra-helical hydrogen bonds stabilize the lateral stacking of twelve a-helices. Assembled under the portal is a head-to-tail (HT) adaptor gp28 ring. This factor is conserved across different phages with low sequence identity^8^. Pa193 HT-adaptor consists of an N-terminal helical core with a C-terminal extension arm used to insert at the portal protomer interface^33^ (Fig. 5b).

As many *Myoviridae* neck and tail assemblies are hexameric^34^, the C6 symmetry map was used to build the Pa193 neck components collar gp29 and gateway gp30 proteins. The collar protein assembles as a 110 Å wide hexamer along with gateway protein, extending the channel formed by the portal:HT-adaptor complex by 100 Å (Fig. 5b). The collar protein contains two extensions, which form a saddle-like interface composed of three loops (residues 28–38; 57–66; 81–86), with the gateway protein (Fig. 5b). Both collar and gateway proteins are rich in β-strands and assemble as a hexameric channel. The gateway protein also contains an α-helical exterior, which is essential for protein:protein interactions with sheath proteins (Fig. 5b).

### Pa193 tail tube and sheath proteins

**Figure 6:**
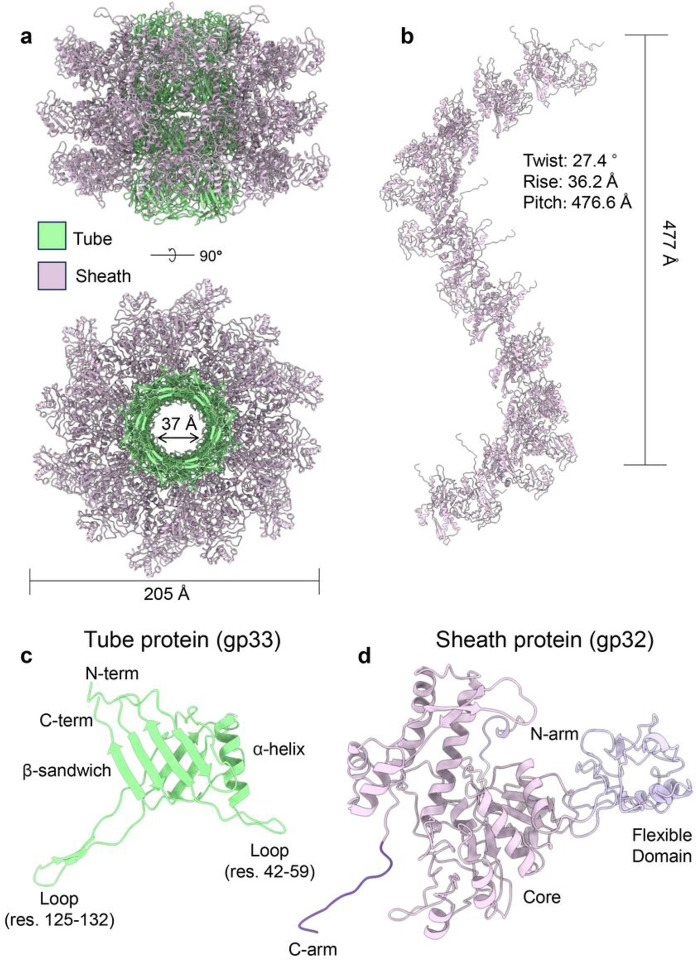
Tertiary and quaternary structure of *Pseudomonas* phage Pa193 tail proteins. (**a**) Side-view of a segment of Pa193 extended tail displayed as cartoon models. Four stacks of hexameric tube subunits (green) form the inner layer of the tail, while three layers of sheath proteins (magenta) assemble around the hexameric tube structure and comprise the outer layer of the tail. (**b**) Sheath protein (12 proteins in one complete turn) comprises a helical tail with a rise of 27.4 Å, a twist of 36.2°, and a pitch of 476.6 Å degrees. (**c**) Cartoon representation of the tail tube gp33 tertiary structure (light green) that comprises a β-sandwich (residues 9–16, 63–68, 97–114, 135–150), backbone a-helix (residues 72–85), and N-term (residues 1–8) extensions. (**d**) Cartoon representation of the sheath protein (gp32) tertiary structure including its flexible domain (residues 91–210), core (residues 23–90; 211–491), C-arm (residues 492–504), and N-arm (residues 1–22).

Most of the particles in the cryo-EM dataset were bacteriophages with extended tails, in stark contrast to the related Pbunavirus E217, which is contracted in about 30% of particles on grid^8^. We determined the cryo-EM reconstructions of the extended Pa193 sheath at 3.5 Å resolution (Fig. 1b, c). We built Pa193 1,300 Å-long extended tail, including 204 copies of tail tube gp33 (residues 1–150) and 204 copies of tail sheath gp32 (residues 1–504)^35^ (Fig. 1a, Fig. 6a). The extended tail contains an internal component composed of 34 stacks of hexameric tube protein gp33 with a ~ 37 Å wide lumen (Fig. 6a, **Supplementary Fig. S4a**). Pa193 sheath protein gp32 comprises a core (residues 23–90, 211–491), a flexible domain (residues 91–210), and N- (residues 1–22) and C-terminal (residues 492–504) extensions used to contact neighboring sheath proteins (Fig. 6d). Pa193 gp32 is similar to the sheath protein of phage E217 (RMSD = 2.3 Å) but different than Pam3 sheath protein, which only contains two domains (RMSD = 20.2 Å) (**Supplementary Fig. S4c d**). Gp32 assembles around the tube structure in a helical arrangement with a pitch of 476.6 Å and a twist and rise of 36.2° and 27.4 Å (Fig. 6b). The tail overall diameter is 205 Å (Fig. 6a); however, the sheath lattice is coarse with openings between subunits, encompassing a ~ 75 Å internal lumen almost entirely occupied by the tail tube gp33 (Fig. 6a). The tail tube protein gp33 consists of a four-stranded antiparallel β-sheet (residues 9–16, 63–68, 97–114, 135–150) facing the exterior of the tail and an α-helix (residues 72–86) that makes intermolecular contact with the sheath (**Supplementary Fig. S4b**). In contrast, gp33 loops spanning residues 42–59 and 125–132 (Fig. 6c) engage in intermolecular interactions with neighboring tube subunits.

### Baseplate proteins

The C6 3.2 Å reconstruction of the Pa193 baseplate has excellent quality (Fig. 1d), allowing us to identify and build 10 polypeptide chains repeated in 66 copies. The ~ 1.4 MDa baseplate complex assembles at the tail end distal to the capsid and comprises three subcomplexes encoded by ORFs 34–47 (Fig. 2).

**Figure 7:**
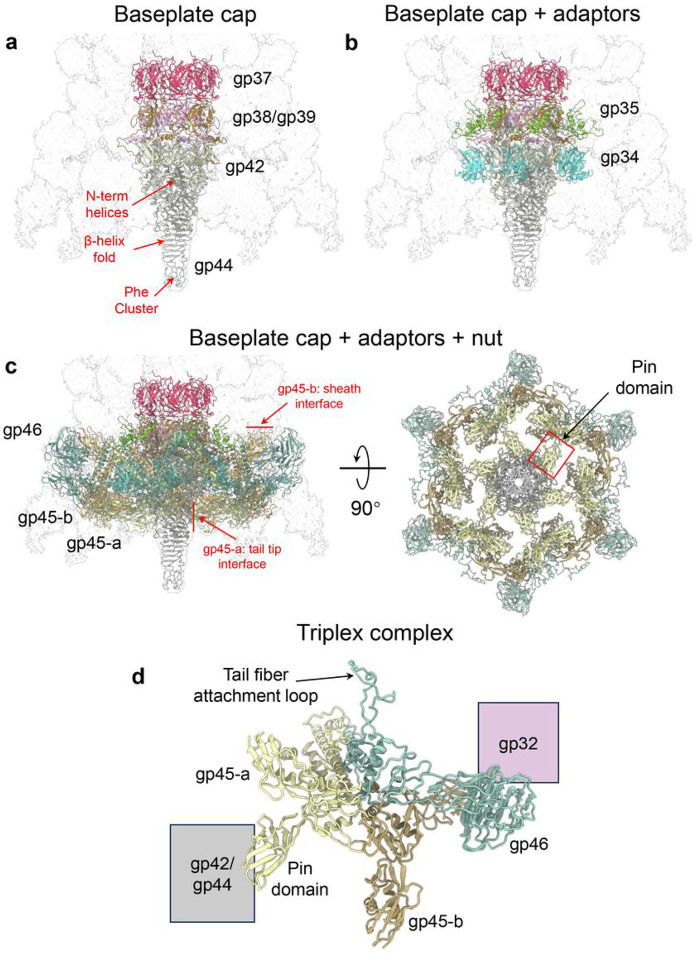
The composition and organization of the simple Pa193 baseplate. (**a**) A semitransparent map of the Pa193 baseplate with overlayed ribbon diagrams of the baseplate cap comprising the baseplate tube gp37, ripcord-1 gp38, ripcord-2 gp39, tail hub gp42, and tail tip gp44. (**b**) The baseplate cap with the addition of two adaptor proteins: the sheath initiator gp35 and helical bundle gp34. (**c**) The baseplate cap, with adaptors and nut complex, comprises six copies of the triplex complexes (Baseplate Wedges gp45-a, gp45-b, and gp46). The left panel shows a bottom-up view of the Pa193 baseplate. The red square shows the position of one gp45-a Pin domain. (**d**) Ribbon diagram of Pa193 triplex complex formed by gp45-a, gp45-b and gp46.

First, the baseplate cap (Fig. 7a), formed by gp37, gp38/gp39, gp42, and gp44, assembles onto the tube and sheath proteins at the Pa193 tail end to seal the tail channel. Gp37 (residues 1–151) has a tertiary structure similar to tail tube gp33 (RMSD 4.2 Å), forming a hexameric ring concentric to the tail tube. Gp37 then provides a platform for gp38 (residues 2–171)/gp39 (residues 1–188) heterotrimers to assemble as another ring concentric to gp37. The heterotrimers generate a 3-fold symmetric complex, which then serves as an attachment point to trimeric gp42 (residues 3–287) and trimeric tail tip gp44 (residues 8–221), sealing the tail (Fig. 7a). The Pa193 tail tip consists of a triple β-helix fold^36^ with three N-terminal α-helices protruding inside the tail interior and a C-terminal Phe cluster generated by F203 and F205 from each chain. This cluster coordinates a discernable globular density, likely a ferric ion (Fe^3+^) also found in analogous contractile ejection systems like Pam3^9^, R-type pyocin^18^, and E217^8^. This trimeric assembly binds the tail hub protein with a 1:1 binding interface.

Second, the Pa193 baseplate includes adaptor subunits gp34 (residues 1–106) and gp35 (residues 2–109) (Fig. 7b). Gp35 binds the outwardly extending C-termini of gp37 and gp38 while gp34 binds tail hub gp42 at residues 11–17; 63–72 and residues 208–214; 153–158 with a 2:1 binding interface. Both gp34 and gp35 decorate the cap complex in six copies that do not make direct contact with each other, suggesting these proteins are adaptors instead of discrete structural components of the baseplate.

The third Pa193 baseplate subcomplex comprises six copies of the triplex complex gp45/gp46 that form a nut-shaped assembly bound to the baseplate bottom (Fig. 7c). Each triplex complex is composed of gp46 (residues 2–504) and two copies of gp45 (residues 2–417) that exists in two different conformations, gp45-a, and gp45-b (Fig. 7d).

The tail is kept in an extended conformation by a network of long-distance bonds between the gp45-a pin domain and the tail tip gp44 (Fig. 7c, left).

In the triplex complex (Fig. 7d), the gp45-a tertiary structure is more globular than gp45-b, which adopts a more extended conformation. The gp45-a:gp46 interface is also smaller than that of gp45-b:gp46 (2,383 Å^2^ vs. 2,678 Å^2^, respectively). Additionally, gp45-b contains a binding interface with the sheath protein, while gp45-a forms a binding interface with the tail hub gp42 and tail tip gp44. Thus, gp45 conformers in the Pa193 baseplate reflect gp45 intrinsic plasticity and differential binding contacts with gp46, tail hub gp42, and sheath gp32.

### Tail Fiber proteins

**Figure 8:**
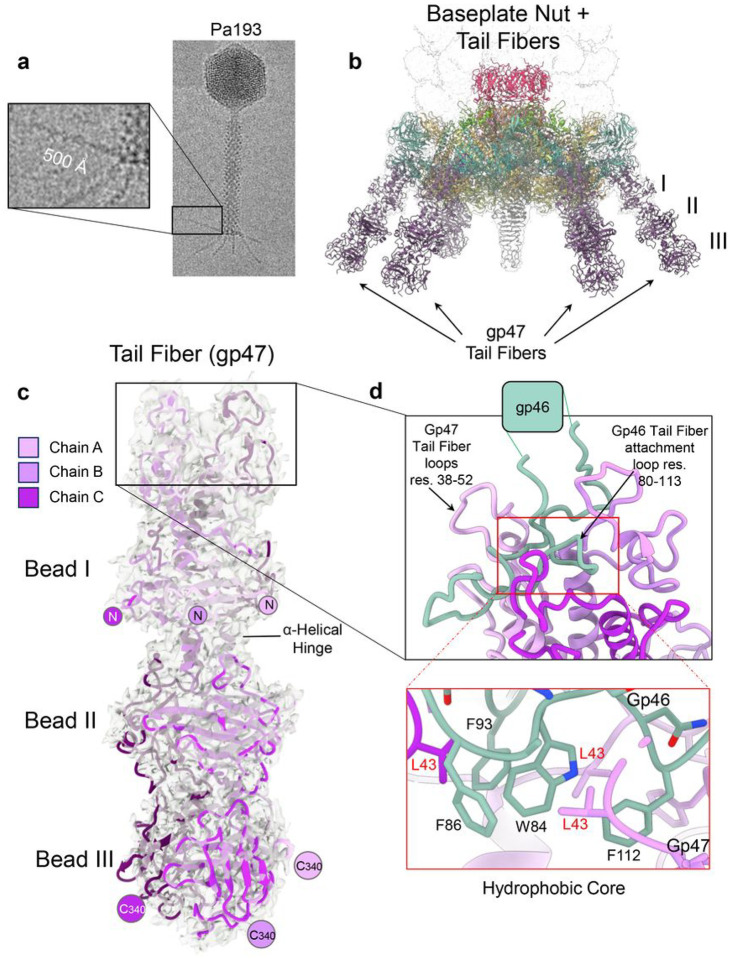
Tail fiber structure and attachment to the baseplate. (**a**) A cryo-micrograph of bacteriophage Pa193 reveals prominent and straight tail fibers. (**b**) Cartoon schematic of the baseplate assembly with the tail fibers (residues 1–340) attached to six vertices of the baseplate. (**c**) Cartoon representation of tail fiber gp47 AlphaFold2 trimeric assembly (residues 1–340) overlaid to the localized reconstruction (calculated at 3.3 Å resolution and displayed at 1.5 σ). (**d**) (Top) Zoom-in view of the interface between baseplate gp46 tail fiber attachment loop (green) and gp47 tail fiber loops (magenta). (bottom) A hydrophobic core formed between gp46 (green) and gp47 (magenta).

Pa193 contains six ~ 500 Å long tail fibers observed in cryo-EM micrographs, surprisingly more rigid than E217 tail fibers^8^ (Fig. 8a). The 3.2 Å baseplate reconstruction revealed density for tail fibers but only up to ~ 150 amino acids (10%) of the tail fiber. A focused reconstruction of the baseplate allowed us to fit an AlphaFold prediction of the tail fiber (residues 1–340), comprising about 30% of the full-length fiber. This model could be subjected to positional refinement, revealing a good fit with the experimental density (CC = 0.71). The overall structure of the tail fiber (residues 1–340) comprises three beads-on-a-string, each consisting of three four-stranded β-sheets (Fig. 8b). Bead I is smaller than beads II and III and contains a triple helix domain, which forms the interface with the baseplate wedges (Fig. 8b). A short α-helical hinge (residues 128–137) connects beads I and II (Fig. 8c), but is missing between beads II and III that are continuous.

We resolved the 3:1 interface between Pa194 gp47 tail fiber loops (residues 38–52) and the triplex complex gp46 tail fiber attachment loop (residues 80–113) (green in Fig. 8d) using the improved quality of the localized reconstruction (Fig. 8d). This binding interface comprises mainly hydrophobic contacts between Trp84, Phe-86/Phe93 and Phe112 (Fig. 8d, **bottom**) in the gp46 tail fiber attachment loop and Ile41, Leu43 from gp47 tail emanating from tail fiber subunits. These hydrophobic residues form an interior core that holds the fibers straight and, thus, is visible in our reconstruction.

### PaP193 tail tip anchors to the C-terminus of the tape measure protein

**Figure 9:**
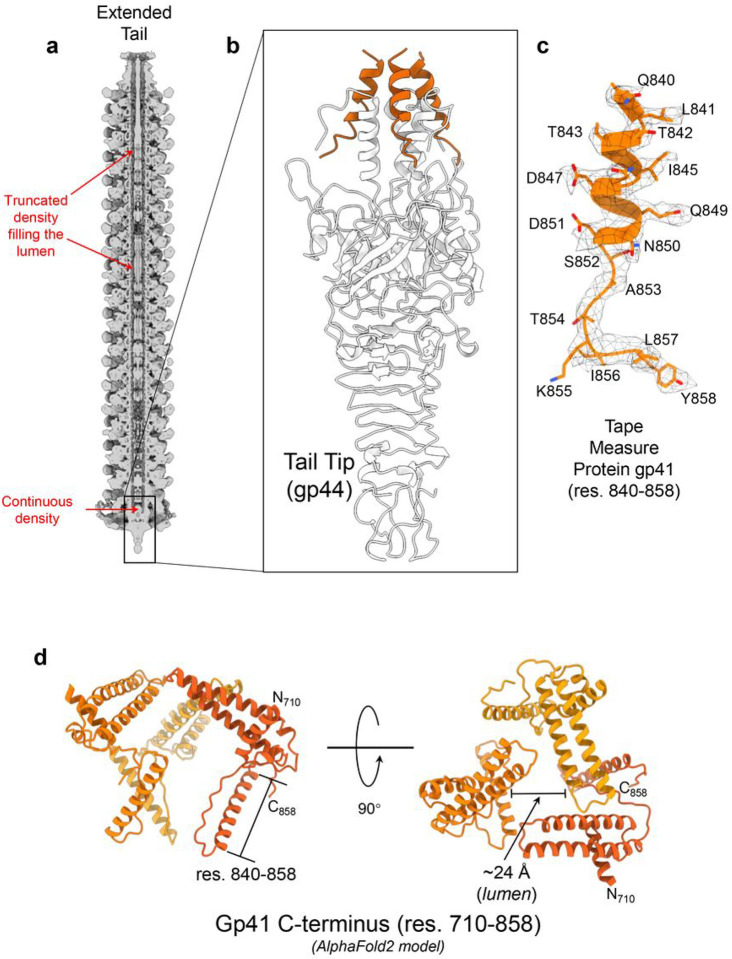
Identification of the Pa193 tape measure protein C-termini. (**a**) Cross-section of Pa193 tail calculated at 7.5 Å resolution and displayed at 3.0 σ. (**b**) Ribbon diagram of the tail tip gp44 (gray) bound to three TMP (gp41) C-termini (orange). (**c**) Zoomed-in view of the trimeric tape measure protein C-term (residues 840–858) overlaid to the C6 reconstruction (calculated at 3.3 Å resolution and displayed at 2.5 σ). (**d**) An AlphaFold2 model of Pa193 TMP C-terminal residues 710–858 predicted as a trimeric assembly. The position of residues 840–858 is shown.

Pa193 tail lumen contains an elongated and truncated density visible at a high contour, suggesting the presence of a macromolecule inside the tail channel (Fig. 9a). The distal end of the tail lumen relative to the phage neck has an expectedly strong and continuous density, looming over the tail tip (Fig. 9b, c). We used Model Angelo to build a model into this density and matched the predicted protein sequence to the C-terminus (residues 840–858) of gp41, which encodes Pa193 tape measure protein (TMP). Gp41 residues visible in the density comprise three a-helices that form a six-helix bundle with the tail tip gp44 (Fig. 9b). The gp41 a-helix contains both hydrophobic (Leu841, Ile845, and Ala848) and acidic (Asp847 and Asp851) residues, and is followed by a C-terminal extended moiety that binds the tail hub gp42 and tail tip gp44 via residues Lys855 and Tyr858 (Fig. 9c). The gp41 moiety visible in the cryo-EM reconstruction shares high sequence homology (100% coverage and >90% identity) to hypothetical TMPs of other contractile-tailed phages in the *Pbunavirus* genus, suggesting a conserved function. Interestingly, an AlphaFold2 prediction of Pa193 TMP C-terminal residues 710–858 (Fig. 9d) suggests a trimeric hollow structure with a ~ 24 Å wide channel, and C-terminal helixes (Fig. 9b, c) pointing away from each other. We hypothesize that the AlphaFold2 model represents a thermodynamically stable conformation of TMP that occurs after the phage has ejected its genome into the host.

## DISCUSSION

Despite the large amount of genomic information in databases and decades of research, *Pseudomonas* phages remain significantly understudied, especially compared to classical model systems that infect Enterobacteriaceae^37^. Annotating *Pseudomonas* phage proteins remains challenging and inherently inaccurate. The lack of structural and functional information has limited the identification and annotation of ORFs in phages of the *Pbunaviruses*, which have biomedical interest in phage therapy^38^. In this paper, we used the power of cryo-EM, localized reconstruction, and conventional proteomics and bioinformatics to annotate 21 structural components of Pa193, a *Pseudomonas Pbunavirus*. This structural atlas led us to uncover three aspects of Pa193 biology that have potential application to other *Myoviridae*.

**Figure 10:**
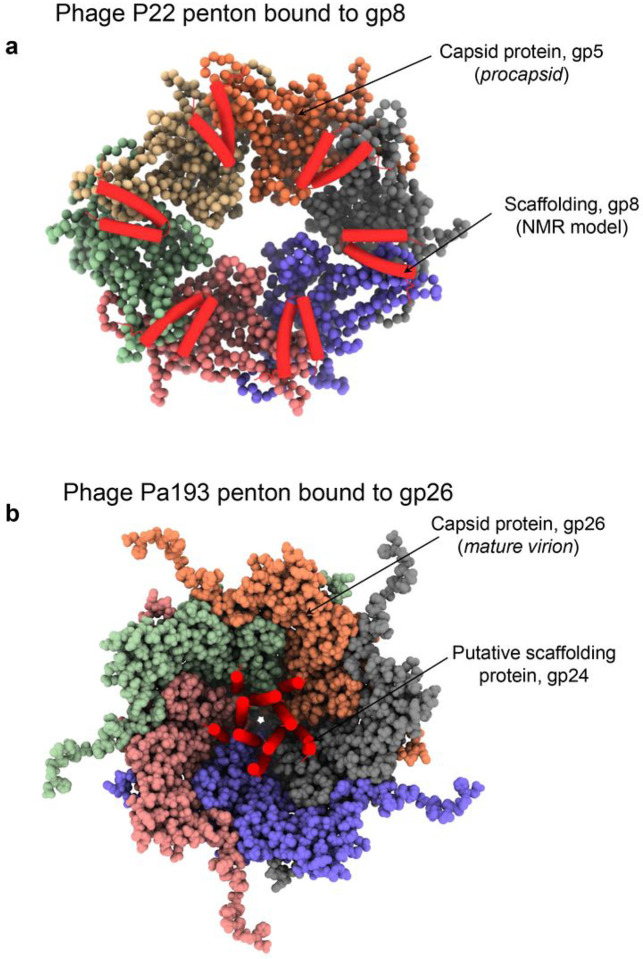
Comparing P22 and Pa193 putative scaffolding proteins. (**a**) A space-filling representation of phage P22 (PDB: 2XYY) (**a**) and Pa193 (**b**) pentons viewed from inside the procapsid and capsid, respectively. P22 scaffolding protein gp8 (PDB: 2GP8) and Pa193 putative scaffolding gp24 are shown as red helical hairpin.

First, we provide evidence that a pentameric helix-turn-helix protein stabilizes the icosahedral 5-fold vertices from the interior of the capsid. A localized five-fold capsid reconstruction revealed a helix-turn-helix identified as the gene product gp24 residues 286–326, possibly consistent with a scaffolding protein. Analysis of the binding interface suggests a mainly electrostatic and polar surface of contact with the capsid interior, again consistent with the nature of a scaffolding protein^39,40^. The presence of this factor in the proteomics analysis suggests that this protein remains in the mature virion. However, based on the current evidence, we cannot determine if gp24 is cleaved or if the rest of the protein remains flexible in the capsid and thus remains invisible in our reconstructions. Also, a similar protein was not seen in the cryo-EM reconstruction of the related *Pseudomonas Pbunavirus* phage E217, which was determined at a comparable resolution^8^. Further, gp24 resembles the tertiary structure of the P22 coat protein-binding domain of the scaffolding protein gp8 (PDB: 2GP8), which forms a helix-loop-helix domain associated with capsid protein at both hexons and pentons^27^. The Chiu lab reported that the P22 procapsid contains a helix-turn-helix-shaped density bound to the capsid interior vertices, thought to be the scaffolding coat protein-binding domain^41^ (Fig. 10a). The fragment of Pa193 gp24 visible in our reconstruction is topologically analogous to the P22 gp8 coat protein-binding domain, even though the two full-length scaffolding proteins have different sizes in P22 and Pa193 (478 versus 303 residues, respectively). Both helix-turn-helix fragments visualized inside the capsid contain a similar binding interface and orient the turn toward the capsid protein A-domain. The P22 scaffolding protein (Fig. 10a) makes electrostatic interactions with the capsid N-terminus^39^ and lies parallel to the plane of the capsid protein, whereas Pa193 gp24 (Fig. 10b) exclusively binds to the capsid protein A-domain, lacks contact with the capsid protein N-terminus and lies perpendicular to the plane of the capsid. However, P22 scaffolding was visualized in the procapsid but not the mature virion^42^, whereas we found gp24 in the Pa193 mature virion, which may account for some of the differences above.

Second, our reconstruction revealed a large portion of Pa193 tail fiber, which folds into a ~ 500 Å elongated trimeric structure with an α-helical coiled-coil hinge near its N-terminus. We annotated and built the first 350 residues of Pa193 tail fiber gp47 and visualized the loops associated with the baseplate triplex complex subunit gp46. Notably, we identified a network of hydrophobic interactions that we hypothesize provide alternative contact points for the side chains of gp46 and gp47, allowing the tail fiber to adopt different binding conformations. This binding mode, similar to that proposed for promiscuous protein binding interfaces^43^, has two advantages. On the one hand, it provides alternative contact points between side chains that increase binding affinity; on the other hand, it retains the flexibility required for the tail fiber to rotate relative to the baseplate akin to a joint.

Third, we identified a C-terminal moiety of the TMP which bears similarities to *Siphoviridae* TMP implicated in genome ejection^44^. Pa193 TMP C-term gp41 residues 840–858 were identified as a trimeric helix that binds the tail tip N-term (gp44), forming a six-helix bundle. This is the likely pre-ejection conformation of TMP which may adopt a distinct quaternary structure after genome ejection^45^. Mounting evidence suggests that the TMP forms a channel through the host cell membrane in *Siphoviridae*, implicated in genome delivery^46^. Cryo-ET studies of phage T5 ejecting its genome into proteoliposomes containing its receptor protein FhuA identified a channel-like structure emanating from the phage tip and penetrating the liposome, thought to be the TMP^47^. TMP involvement in genome ejection was also suggested for phage λ, where the TMP can extrude from the phage tail and associate with LamB-decorated liposomes *in vitro*^48^, allowing ions to traverse the liposome membrane^49^. Similarly, in HK97, delivery of the phage genome requires TMP the IM glucose transporter protein, PtsG, and the periplasmic chaperone, FkpA^44^. However, less is known about the involvement of TMP in *Myoviridae* genome delivery, which is driven by a contraction-coupled ejection of DNA^34^. Cryo-ET studies in T4 revealed that, during infection, the phage binds bacterial receptors, and sheath contraction leads to the piercing of the OM by the tail tip, leading to a displacement relative to the host OM^17^; however, the tail does not appear to penetrate the IM but stops at the PG layer^17^. A PG hydrolase is then ejected from the phage, identified as a domain of the TMP in some phages^50^, to degrade the host cell wall. Accordingly, we detected a putative transglycosylase domain between residues 491 and 582 of Pa193 gp41. In some *Myoviridae* phages, the TMP contains a helical structure with disordered regions flanking a helical domain that forms an elongated coiled-coil structure^9^. Upon host attachment and membrane penetration, ejection of the tail tip leads to concomitant ejection of the TMP C-term, which forms a platform for folding in the periplasm into a PG hydrolase and then a channel used as a genome conduit. We hypothesize that Pa193 gp41 is elongated and unfolded in the channel before ejection but folds upon release into the host to form a channel with its C-terminus. An AlphaFold2 prediction of the gp41 C-terminus (Fig. 9d), which likely captures the post-ejection conformation of the protein, supports this idea. The proposed role of Pa193 TMP as a *de facto* ejection protein^45^ is supported by direct and indirect evidence in *Siphoviridae* but is new to *Myoviridae*.

In summary, we have deciphered the architecture and design principles of Pa193, the second *Pbunavirus Pseudomonas* phage whose structure has been thoroughly annotated, from head to baseplate, using cryo-EM SPA analysis. The results of this study expand the repertoire of *Pseudomonas* structures solved at atomic resolution, providing valuable information to decipher differences in phage specificity, stability, and resistance mechanism. The 3D-atlas of Pa193 structural proteins described in this paper will support the mapping of mutations altering phage functionality and the rational optimization of phages with potential phage therapy applications.

## MATERIALS AND METHODS

### Origin and characteristics of Pa193

Pa193 was isolated from sewer samples of the greater Sydney metropolitan area, Australia. Pa193 was part of a phage cocktail candidate developed by Armata Pharmaceuticals. Fermentation and purification were achieved using proprietary methods in order to achieve clinical levels of purity and a titer of 1E13 PFU/ml. Pa193 genome was published under accession number NC_050148.1. The annotations were revised prior to the initiation of this work, and an updated annotation table is provided in **Supplementary Table S2**.

### Vitrification and data collection

2.5 μL of virions, measured at a PFU of 1 × 10^13^ phages/mL, was applied to a 200-mesh copper Quantifoil R 2/1 holey carbon grid (EMS) previously glow-discharged for 60 sec at 15 mA using an easiGlow (PELCO). The grid was blotted for 7.5 sec at blot force 2 and vitrified immediately in liquid ethane using a Vitrobot Mark IV (Thermo Scientific). Cryo-grids were screened on 200 kV Glacios (Thermo Scientific) equipped with a Falcon4 detector (Thermo Scientific) at Thomas Jefferson University. EPU software (Thermo Scientific) was used for data collection using accurate positioning mode. For high-resolution data collection of the Pa193, micrographs were collected on a Titan Krios (Thermo Scientific) microscope operated at 300 kV and equipped with a K3 direct electron detector camera (Gatan) at the National Cryo-EM Facility at the Pacific Northwest Cryo-EM Center, (PNCC).

### Liquid chromatography/mass spectrometry (LC/MS/MS) analysis

Phage samples were treated with 12 mM sodium lauryl sarcosine, 0.5% sodium deoxycholate, and 50 mM triethyl ammonium bicarbonate (TEAB), heated to 95°C for 10 min and then sonicated for 10 min, followed by addition 5 mM tris(2-carboxyethyl) phosphine and 10 mM chloroacetamide to fully reduce, and alkylate the proteins in sample. The sample was then subjected to trypsin digestion overnight (1:100 w/w trypsin added two times). Following digestion, the sample was acidified, lyophilized, and then desalted before injection onto a laser-pulled nanobore C18 column with 1.8 μm beads. This was followed by ionization through a hybrid quadrupole-Orbitrap mass spectrometer.

Most abundant proteins were identified by searching the experimental data against a phage protein database, pseudomonas host protein database, and a common contaminant database using the MASCOT algorithm^51^.

### Cryo-EM SPA

All *Pseudomonas* phage Pa193 datasets were motion-corrected with MotionCorr2^52^. RELION’s implementation of motion correction was applied to the micrographs with options of dose-weighted averaged micrographs and the sum of non-dose weighted power spectra every 4 e^−^/Å^2^. CTF (Contrast Transfer Function) was estimated using CTFFIND4^53^. After initial reference picking and 2D classification, particles were subjected to a reference-free low-resolution reconstruction without imposing symmetry. The particles were then 3D and classified into four classes, with I4 symmetry imposed. Of the four classes, the best class was chosen and was subjected to 3D auto-refinement to align the particles finely. The particles were then expanded according to I4 symmetry using RELION’s *relion_particle_symmetry_ expand* function to obtain 60 times the initial particles. A cylindrical mask (r = 200 Å) was generated using SCIPION 3.0^54^ and then resampled onto a reference map covering the five-fold vertex in Chimera^55^. The cylindrical mask was then used for non-sampling 3D classification without imposing symmetry to search for the tail. Locally aligned particles were then combined, and duplicate particles were removed. The initial localized reference map was reconstructed directly from one of the classes using RELION’s *ab initio* 3D Initial Model. Selected 3D classes were auto-refined using C5 symmetry, followed by five-fold particle expansion. The expanded particles were subjected to a third 3D classification, and the map was symmetrized by imposing C12 and C6 symmetries, which gave the best density for the portal: head-to-tail and the collar: gateway: tube: sheath protein complexes, respectively. All steps of SPA, including 2D Classification, 3D classification, 3D refinement, CTF refinement, particle polishing, post-processing, and local resolution calculation, were carried out using RELION 3.1.2^56,57^. The final densities were sharpened using *phenix.autosharpen*^58^. RELION_*posprocess*^56,57^ was used for local resolution estimation. All cryo-EM data collection statistics are in Table 1. A pipeline of SPA is shown in **Supplementary Fig. S1, S2.**

### De novo model building, oligomer generation, and refinement

The 3.2 Å C12-averaged localized reconstruction^59^ was used to build a model of dodecameric portal protein bound to twelve copies of the head-to-tail. Pa193 portal was modeled between residues 94–528, while C-terminal residues 529–765 had no discernable density. The full-length head-to-tail protein (residues 1–155) was built in the C12 map. Using the 3.5 Å C6 localized reconstruction, we built a complete model of the hexameric collar (residues 1–132) bound to six copies of the gateway (residues 1–183). The tube (residues 1–150) and sheath (residues 1–504) proteins were also built using the 3.5 Å C6 map. We generated and used an AlphaFold2 model of full-length sheath protein gp32 and rigid body fit, and we refined this model in PHENIX. We used the 3.2 Å C6 baseplate reconstruction to build the following proteins: helical bundle, sheath initiator, baseplate tube, ripcord-1, ripcord-2, tail hub, tail tip, baseplate wedge 1, and baseplate wedge 2. We rigid body fit an AlphaFold v2 prediction of the trimeric tail fiber (residues 1–340) into the 3.3 Å local map with a map-to-model CC of 0.71. At the last refinement step, the 3.3 Å localized reconstruction of the baseplate was used to refine all baseplate models, including the tail fiber (residues 1–340). All *de novo* atomic models shown in this paper were built manually using Coot^60^ or Chimera^55^ and refined using several rounds of rigid-body, real-space, and B-factor refinement using *phenix.real_space_refmement*^61^. The final models of the capsid: cementing (19 chains), portal: head-to-tail adaptor (24 chains), collar: gateway: sheath: tube: (54 chains), and helical bundle: sheath initiator: baseplate tube: ripcord-1: ripcord-2: tail hub: tail tip: baseplate wedge 1: baseplate wedge 2 (48 chains) yielded a final Correlation Coefficient (CC) of 0.87, 0.86, 0.84, and 0.80 respectively, and excellent stereochemistry (Table 2).

### Structural analysis

All ribbon and surface representations were generated using ChimeraX^62^. Drawings of electron density maps and local resolution maps were generated using ChimeraX^62^. Structural neighbors and flexible regions were identified using the DALI server^63^. Binding interfaces were analyzed using PISA^64^ and PDBsum^65^ to determine bonding interactions, interatomic distances, and types of bonds. The Coulombic Electrostatic Potential was calculated and displayed with surface coloring using ChimeraX^62^. The helical parameters of the extended tail were measured using HI3D^66^.

## Figures and Tables

**Table 1:** Cryo-EM data collection statistics

Data Collection	*Pseudomonas* Phage Pal 93
**Facility/Microscope**	PNCC/Krios
**Camera**	K3
**Voltage (kV)**	300
**Magnification**	64,000 X
**Electron exposure (e** ^**-**^ **/ Å** ^**2**^ **)**	34.4
**Defocus range (pm)**	−0.75 to − 1.75
**Pixel Size (Å/px)**	0.67
**Total movies (frames/movie)**	12,520 (52)
**Initial particle no.**	46,075
**Final particle no. (symmetry)**	9,179 (C6)

**Table 2:** Models Statistics

PDB entry code		9B40	9B41	9B42	9B45
**Symmetry**		C5	C12	C6	C1
**Model subunits**		Major Capsid: Decorating: *putative* Scaffolding	Portal: Head-to-Tail Adaptor	Collar: Gateway: Tube: Sheath	Baseplate Proteins
**Map resolution (Å)**		2.9 Å	3.2 Å	3.5 Å	3.3 Å
**Fourier Shell Correlation threshold**		0.143	0.143	0.143	0.143
**Initial model used (PDB code)**		*de novo*	*de novo*	*de novo*	*de novo*
**Map-to-Model Correlation Coefficient (CC)**		0.87	0.86	0.84	0.80
**Model Composition**	**Number of Chains**	19	24	19	54
	**Nonhydrogen Atoms**	32,946	55,608	25,948	104,307
	**Residues**	4,325	7,080	3,294	13,848
**RMS deviations**	**Bond lengths (Å)**	0.004 (0)	0.005 (0)	0.006 (0)	0.004 (1)
	**Bond angles (degrees)**	0.7 (8)	1.1 (5)	0.9 (9)	0.8 (54)
**Validation**	**MolProbity Score**	1.9	1.8	2.2	2.3
	**Clash Score**	3.8	6.7	11.7	17.8
	**Rotamer Outliers**	3.8	0.9	1.6	0.7
**Ramachandran plot**	**Favored**	95.8%	94.2%	91.8%	90.2%
	**Allowed**	4.1%	5.7%	8.0%	9.1%
	**Outliers**	0.1%	0.0%	0.3%	0.7%

## Data Availability

The atomic models and three-dimensional reconstructions described in this paper are available in the Protein Data Bank (9B40, 9B41, 9B42, 9B45) and Electron Microscopy Data Bank (44163, 44164, 44166, 44168), respectively. All other data are available from the corresponding author on reasonable request.
